# Tuning Infrared Plasmon Resonance of Black Phosphorene Nanoribbon with a Dielectric Interface

**DOI:** 10.1038/s41598-018-21365-2

**Published:** 2018-02-19

**Authors:** Desalegn T. Debu, Stephen J. Bauman, David French, Hugh O. H. Churchill, Joseph B. Herzog

**Affiliations:** 1 University of Arkansas, Department of Physics, Fayetteville, Arkansas 72701 USA; 2 University of Arkansas, Microelectronics-Photonics Graduate Program, Fayetteville, Arkansas 72701 USA

## Abstract

We report on the tunable edge-plasmon-enhanced absorption of phosphorene nanoribbons supported on a dielectric substrate. Monolayer anisotropic black phosphorous (phosphorene) nanoribbons are explored for light trapping and absorption enhancement on different dielectric substrates. We show that these phosphorene ribbons support infrared surface plasmons with high spatial confinement. The peak position and bandwidth of the calculated phosphorene absorption spectra are tunable with low loss over a wide wavelength range via the surrounding dielectric environment of the periodic nanoribbons. Simulation results show strong edge plasmon modes and enhanced absorption as well as a red-shift of the peak resonance wavelength. The periodic Fabry-Perot grating model was used to analytically evaluate the absorption resonance arising from the edge of the ribbons for comparison with the simulation. The results show promise for the promotion of phosphorene plasmons for both fundamental studies and potential applications in the infrared spectral range.

## Introduction

Studies of the light-matter interaction have been conducted for many materials, commonly focusing on noble metal films and nanostructures. Noble metals (gold, silver, etc.) support strong surface plasmon confinement. Surface plasmons are collective wave modes of conduction band electron oscillations at the interface between two materials; the waves are coherent with an incident oscillating electromagnetic field^1–4^. Strong locally confined fields can lead to enhanced light absorption and Raman scattering. The response of plasmonic metals is predominantly limited to the spectral range from ultraviolet to near-infrared (NIR). Beyond this spectral range, plasmons generate weak field confinement, have narrow spectral resonance due to large negative permittivity^5^, and exhibit very limited tunability due to high losses^4–6^.

As an alternative, two-dimensional (2D) materials such as graphene have demonstrated low attenuation of surface plasmon resonance, which is attributed to a unique band structure and high carrier mobility^7,8^. Graphene plasmons typically occur for the spectral range spanning mid-IR to low terahertz^8,9^. This has been achieved experimentally through a wide range of tuning mechanisms – higher doping levels, reduced structure dimensions, and gate modulation^9–12^. Fabricating graphene requires a substrate and a dielectric environment, which causes the plasmon-phonon modes to split into two hybrid modes. The dielectric environment surrounding graphene plasmonic structures can also cause weak dispersion and short lifetimes. The coupling between phonons and the plasmon damping effect hinder the utilization of graphene for enhanced light absorption in the low terahertz to mid-IR range^12–14^.

Very recently, black phosphorus (BP), a layered semiconductor with a two-dimensional “puckered” hexagonal structure in each monolayer (known as phosphorene), has gained attention in the scientific community as a potential candidate to study surface plasmon polaritons^15–20^. Theoretical and simulation results have revealed that properties of black phosphorous surface plasmons include polarization dependence when exposed to an electromagnetic field^21,22^, dependence on the size of the monolayer^23^, a quantized magnetic field indicated by discretized anisotropic magneto-excitons^24^, and damping point defects and potential for long-range disorder^25^. These features are attributed to its high mobility and highly tunable, layer-dependent, direct bandgap (0.3 eV in bulk to 2 eV in a monolayer)^26–28^, as well as its highly anisotropic in-plane electronic and optical properties^28^. In addition, these desirable properties make BP suitable for other optical material applications such as hyperspectral imaging, thermal imaging, photodetectors in silicon photonics, and terahertz transistors^29–32^. To date, these opto-electronic properties have been limited to fewer applications due to the instability of BP in ambient conditions^33–37^.

In this paper, we explore BP as an alternative 2D material to address the challenges faced by metals and graphene for surface plasmon resonance responses to incident light in the mid- to far-infrared spectral range. First, we focus on a theoretical analysis of the dispersion relation and the confinement strength of surface plasmon modes excited by a linearly-polarized plane wave on an infinite phosphorene sheet, taking the surrounding dielectric media into account. Next, we expand the theoretical work to periodic monolayer BP nanoribbons using finite element simulations. We select a design that can be easily realized in experiments and use numerical simulations to describe the tunable resonance and enhanced absorption of the plasmonic modes for capping layers and substrates of different dielectric values. Further, a theoretical periodic grating model is implemented to determine the wavelength of the resonant absorption peak by calculating the phase of the reflected wave at the edge of the nanoribbon. We also extend the numerical simulation to study BP nanoribbons enhancing absorption in different directions based on the optical conductivity change and ribbon width. Finally, we study mechanisms of preserving phosphorene from oxidation effects while maintaining edge plasmon enhanced absorption. Although phonon-related damping pathways for BP plasmons remain unknown, this work highlights several attractive features of tunable mid- to far-infrared BP plasmons.

## Results

### Black Phosphorene Conductivity Model

Anisotropic, angular frequency (*ω*) dependent, dynamical, 2D surface local conductivity of BP can be described by the semi-classical Drude model expression^21^.

1 $${\sigma }_{jj}=\frac{i\hslash {D}_{j}}{\pi (\hslash \omega +i\eta )}$$

Here, *j* denotes the position along the arm-chair direction (*x*) and zigzag direction (*y*). *D*_*j*_ = *πNe*^2^/*m*_*j*_ is the Drude weight, which is dependent on the electron charge, *e*, anisotropic effective electron mass, *m*_*j*_, and electron density, *N*. *η* = 1/*τ* is the scattering rate (*τ* is the carrier relaxation time, related to finite damping.) The anisotropic effective mass for monolayer or bulk BP gives rise to anisotropic conductivity. Along a plane near the *Γ*-point in the BP band diagram, the effective electron mass along the *x*- and *y*-directions are ${m}_{cx}={\hslash }^{2}/(\frac{2{\gamma }^{2}}{{\rm{\Delta }}}+{\eta }_{c})$ and ${m}_{cy}={\hslash }^{2}/2\,{v}_{c}$^21,38^. Values of the conduction band parameters for monolayers include the following: ${\eta }_{c}={\hslash }^{2}/0.4\,{m}_{0}$, ${v}_{c}={\hslash }^{2}/1.4\,{m}_{0}$, ${\rm{\Delta }}\,=2\,{\rm{eV}}$, and *γ* = 4*a*/*π* eVm, where *a* = 0.223 nm and *π*/*a* is the width of the Brillouin Zone in the *x*-direction^21,38^. These are chosen such that they yield the known conduction band effective masses *m*_*cx*_ ≈ 0.15 *m*_0_ and *m*_*cy*_ ≈ 0.7 *m*_0_ of monolayer BP. It is worth mentioning that the band parameters are highly sensitive to the number of BP layers; any small change explicitly affects anisotropic effective masses^38^. We choose the electron carrier density to be from *N* = 10^12^ to 5 × 10^13^ cm^−2^, and a scattering rate of *η* = 10 meV that accounts for the finite damping^21^. These *N* values are within the range reported in *ab initio* studies, giving this scattering rate^39,40^. Monolayer phosphorene is an ultra-thin film with a thickness of *t*_*BP*_ ≈ 0.7 nm^27^. Although the monolayer thickness extracted from bulk black phosphorus is 0.5 nm^39^, we choose a slightly larger value consistent with the measured height of most samples^18^ because this is the one most likely to be realized in experiments and the one that will determine observed plasmonic effects. We can introduce a phosphorene layer with volumetric anisotropic permittivity^41^ converting the 2D surface conductivity into a 3D conductivity using the relation *σ*_2*D*_ = *t*_*BP*_*σ*_3*D*_^42^. Hence, the 3D complex anisotropic dielectric function for monolayer BP from Eq. (1) is

2 $${\varepsilon }_{jj}={\varepsilon }_{r}+\frac{i{\sigma }_{jj}}{\omega {\varepsilon }_{0}{t}_{BP}}$$

where *ε*_*r*_ = 5.65 is the relative permittivity for monolayer BP^43^. This approach has been previously used in the investigation of surface plasmons in BP^23^ and graphene 2D films and nanoribbons^44^.

**Figure 1:**
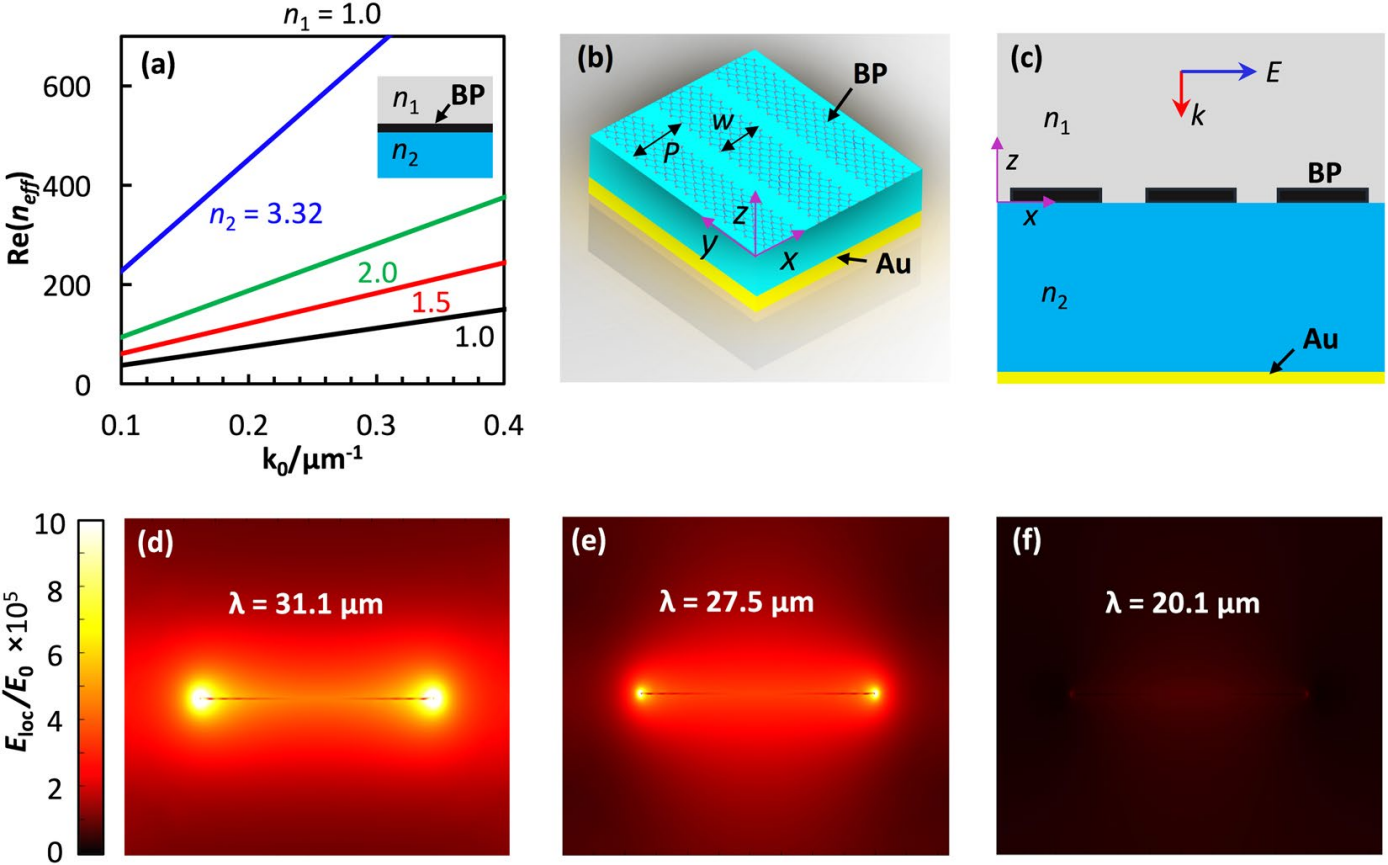
Simulation of the electromagnetic response of illuminated BP. **(a)** Plot of the real part of the SPP modes supported by a BP infinite sheet for four different dielectric media as measured by its vacuum wave vector. **(b)** 3D schematic of periodically patterned phosphorene nanoribbons on a dielectric layer (light blue) atop a gold reflective surface. Period, *P*, and ribbon length, *w*, are labeled. **(c)** Cross-sectional view of **(b)** with the BP, Au, two dielectric layers, *n*_1_ and *n*_2_, and light propagation and polarization directions, *E* and *k*, labeled. **(d**–**f)** Calculated distributions of the electric field enhancement, which is defined as the ratio of the local electric field amplitude, *E*_*loc*_, to that of the incident light, *E*_0_. The modeled parameters include *w* = 15  nm, *P* = 250 nm, *n*_1_ = 1.0, and *n*_2_ = 1.71, *N* = 10^13^ cm^−2^, at *λ* = **(d)** 31.1, **(e)** 27.5, and **(f)** 20.1 µm.

The theoretical dispersion calculation of the plasmonic wave for the transverse magnetic (TM) and transverse electric (TE) modes in a continuous BP monolayer was performed following the method outlined by Ju *et al*. and Grigorenko *et al*.^45,46^. A BP layer is situated in the *x*-*y* plane, sandwiched between semi-infinite dielectric materials of relative permittivity *ε*_1_ (above) and *ε*_2_ (below). Accordingly, calculations for TM mode and TE mode, propagating perpendicular to the interface between the dielectric medium, are

3a $${\varepsilon }_{1}/{k}_{z1}\,+\,{\varepsilon }_{2}/{k}_{z2}\,=\,-i{\sigma }_{jj}/{\varepsilon }_{0}\omega \,$$

3b $${k}_{z1}\,+\,{k}_{z2}=\,i{\sigma }_{jj}{\mu }_{0}\omega \,$$

where *ε*_0_ is the vacuum permittivity, *μ*_0_ is the vacuum permeability, ${k}_{z1}=\,\sqrt{{k}_{jj,1}^{2}-{\varepsilon }_{1}{k}_{0}^{2}}$ and ${k}_{z2}=\,\sqrt{{k}_{jj,2}^{2}-{\varepsilon }_{2}{k}_{0}^{2}}$ are wave vectors above and below the BP layer and *k*_0_ is the vacuum wave vector. A rough measure of the plasmon mode confinement comes from the real part of Eq. (3a) and (3b). This can be attained when ${k}_{jj,1}^{2}\gg {\varepsilon }_{1}{k}_{0}^{2}$ and ${k}_{jj,2}^{2}\gg {\varepsilon }_{2}{k}_{0}^{2}$, leading to *k*_*z*1_ = *i*(*ε*_1_ + *ε*_2_)*ε*_0_*ω*/*σ*_*jj*_ for the TM mode and *k*_*z*1_ = *iσ*_*jj*_ *μ*_0_*ω*/2 for the TE mode. Accordingly, the surface plasmon confinement factor, *n*_*eff*_, for the infinite BP sheet is related to the free-space wave vector by *n*_*eff*_ = *k*_*z*1_/*k*_0_. The real part of *n*_*eff*_ is directly related to the degree of confinement, and the imaginary component corresponds to the propagation length. Figure 1(a) plots analytical results of TM mode light dispersion for four selected dielectric substrates with *n*_1_ taken to be air. The confinement strength indicates a directly proportional effect of the dielectric constant of the materials surrounding the infinite BP layer. The plasmon confinement strength is on the order of a hundred over the IR range, being comparable to that of graphene^47^, while the value for noble metals is close to one. It can be noted that TE mode confinement is barely possible, as the imaginary part of the conductivity, *σ*_*jj*_, in Eq. (3b) is positive over the infrared range of the spectrum, corresponding to high loss. It is worth noting that the surface plasmon dispersion can be controlled by the optical conductivity of phosphorene via *N*. Doing so enables switching between surface plasmon modes that are strongly IR-supported and those that are not.

An alternate way to realize strong coupling and extreme field confinement with localized plasmons is by decreasing a BP sheet to finite nanoscale in-plane dimensions^23^. Finite size BP can add exotic edge states and lateral confinement in the main band gap^48^. For ideal edges and sub 10 nm scale structures, these would need to be addressed to take into account the quantum effects on the plasmonic resonance. However, atomic resolution scanning tunneling spectroscopy of exfoliated black phosphorus reveals only a trivial modification of the band gap at the sample edges^49^. In addition, monolayer black phosphorus nanoribbon widths below a few nanometers are required to significantly modify the band gap^48,49^. In this study we focus on properties of plasmonic responses for periodic nanoribbons with a minimum width of 100 nm and minimum gap of 25 nm. Due to this geometry, the spectral range of the plasmonic resonances studied here is substantially greater than the Fermi wavelength of BP nanoribbons^48^. Because of the aforementioned conditions, the quantum effects can be neglected. From here forward, we focus on properties of plasmonic responses for such reduced periodic arrays of BP ribbons. We have investigated these using a classical model with optical constants of infinite 2D monolayers of black phosphorus, Eq. (1) and Eq. (2).

A 3D schematic view of the structure designed for the study of localized surface plasmon polaritons (LSPPs) supported by BP is depicted in Fig. 1(b), with a corresponding 2D cross-sectional view shown in Fig. 1(c). The arrays of monolayer BP nanoribbons are periodically arranged in the *x*-*y* plane (z = 0). To confine the enhanced light, an optically thick gold reflector surface was added to the bottom of the model. In the z-direction, nanoribbons are separated from this reflector surface by a dielectric spacer with refractive index *n*_2_ = (*ε*_2_)^1/2^, (z < 0). A top dielectric medium with refractive index *n*_1_ = (*ε*_1_)^1/2^, (z > 0), covers the BP nanoribbon arrays. Data from Palick *et al*. for wavelength-dependent optical constants of gold were applied to the simulation^50^.

The electric field intensity distributions obtained from a finite element electromagnetic simulation^51^ are shown in Fig. 1(d–f) for the simulated illumination via plane wave at downward normal incidence for three different wavelengths. The width (*w*) of the BP ribbon was set to 150 nm, the period (*P*) to 250 nm, and the gap (*g*) between each ribbon was *g* = *P* − *w* = 100 nm. The ribbon width and period were selected so that the tunable range for wavelength went through the far-IR region of the electromagnetic spectrum. The ribbon was modeled to be surrounded by air (*n*_1_ = 1.0) on the top surface and a dielectric substrate (*n*_2_ = 1.71) of thickness 5 µm beneath. The dielectric substrate was made greater than *λ*/2 to avoid any coupling effects of the local fields near the BP ribbon and the gold surface. The field distribution reveals that the surface plasmon is highly confined at the edges of the nanoribbon and the confinement strength of the localized field is highly dependent on the excitation wavelength.

**Figure 2:**
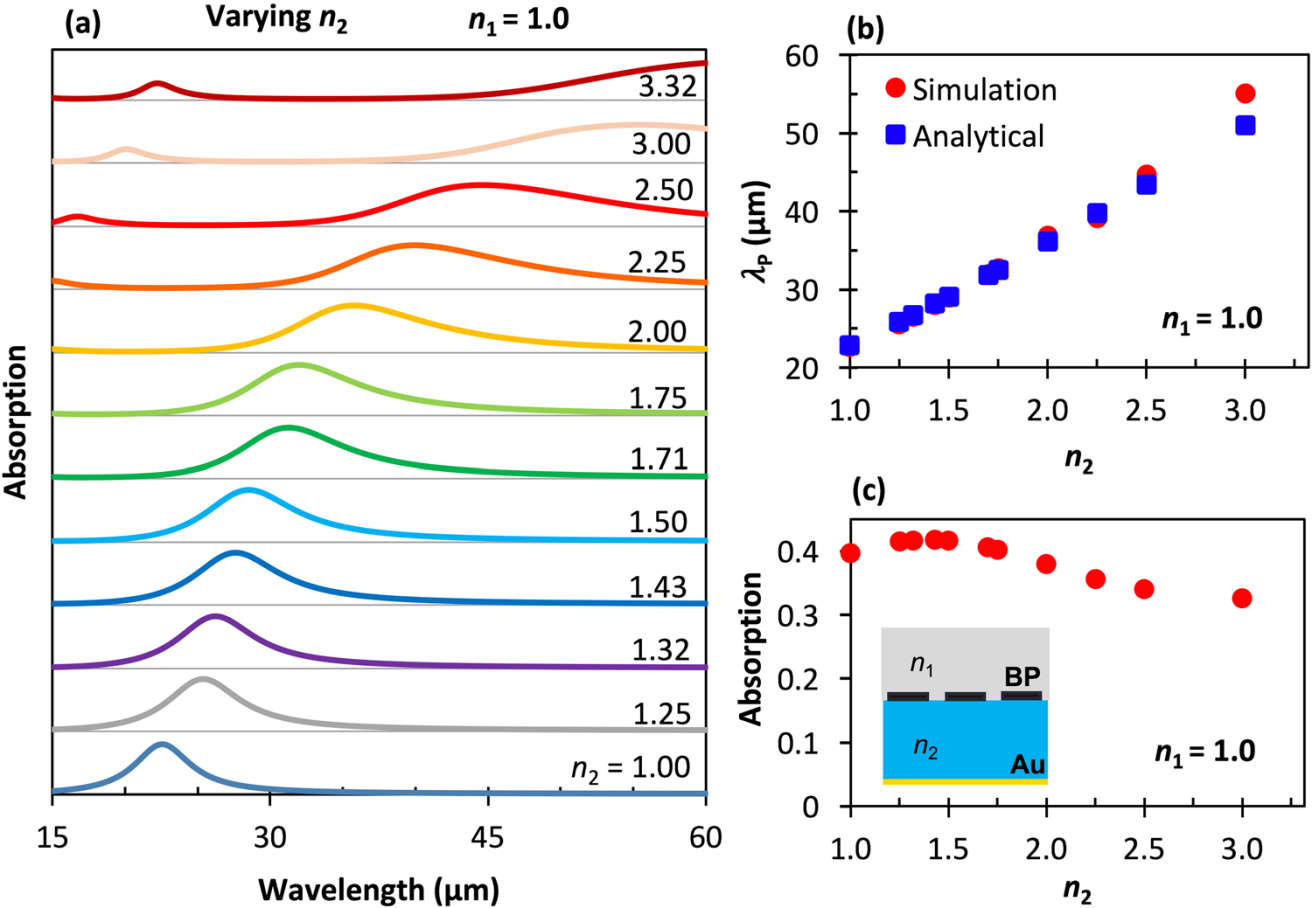
Calculated results showing the effects of varying *n*_2_ on the absorption spectrum with *n*_1_ held constant. **(a)** Simulated normal-incidence TM mode electric field absorption spectra for BP nanoribbons surrounded by air (*n*_1_ = 1.0) and different substrate materials with refractive indices, *n*_2_, and a BP electron density of *N* = 10^13^ cm^−2^. **(b)** Absorption peak resonant wavelength of the fundamental mode (*m* = 1) with respect to the refractive index of the substrate dielectric layer (*n*_2_). The red dots are from the finite-element method (FEM) simulations, and the blue dotes are calculated from the theoretical model described in Eq. (4). (**c**) Peak absorption amplitude as a function of *n*_2_.

Strong field enhancement and localization of plasmon modes in the two-dimensional structure leads to enhanced spectral absorption depending on the shape and the selection of appropriate surrounding dielectric material^52^. Figure 2(a) displays simulated normal-incidence absorption spectra of the BP nanoribbons for *w* = 150 nm and *P* = 25  nm. Here, the top medium was set to the refractive index, *n*_1_, of air, and the absorptive substrate was swept from *n*_2_ = 1.0 to 3.32. Some of the selected indices of refraction, *n*_2_, values chosen match materials such as Al_2_O_3_ (1.71)^53^, KBr (1.43)^54^, PMMA (polymethyl methacrylate, 1.45^55^), PS (polystyrene, 1.50)^55^, and Si (3.32)^50^. Figure 2(b) is a plot of the resonant wavelengths (those with absorption peaks) versus the *n*_2_ values at which they occur, and Fig. 2(c) shows the absorption values at these peaks.

As *n*_2_ is increased, the absorption peak position shifts to higher infrared wavelengths, Fig. 2(b), and broadens; and the amplitude generally decreases, as plotted in Fig. 2(c), indicating increased damping. Following grating theory, the peak absorption wavelengths, matching the resonant conditions of periodic BP nanoribbon, can be obtained from Eq. (4) under irradiation of light at normal incidence,

4 $${\lambda }_{p}=\pi c\sqrt{\frac{2P{\varepsilon }_{0}({\varepsilon }_{1}+{\varepsilon }_{2}){m}_{j}}{m\pi N{e}^{2}}}$$

Here, *λ*_*p*_ is the resonance wavelength of the BP plasmons, *c* is the speed of light, *P* is the period, *ε*_0_ is free space permittivity, *e* is the charge of a single electron, and *m* is a positive integer (*m* = 1, 2, 3, …) representing the order of the dispersion of the mode confinement diffraction. The resonance wavelengths of the analytical solution, Eq. (4), obtained via the periodic grating approach, do not consider the near-field interaction between the BP ribbons when the period is much smaller than the surface plasmon resonance wavelength, and disregards nonlocal effects. Due to coupling of plasmon waves between nearby BP nanoribbons and multiple anomalous reflections between the two edges of the ribbon, the reflected plasmon waves can form an interference process that incorporates a phase factor other than *π*. The surface plasmon at the resonance point undergoes constructive interference with the reflected wave between the edge, satisfying 2 *wRe*(*k*_*z*1_) + 2*ϕ* = 2 *mπ*^56,57^. Here, *w* is the ribbon width, *ϕ* is the reflection phase at the edge, and *m* is an integer for the peak resonance order. The value of *ϕ* can be obtained analytically by fitting the simulated data of a given ribbon surrounded by an arbitrary dielectric medium using Eq. (5),

5 $$\varphi /\pi =m-\,\frac{{\varepsilon }_{0}({\varepsilon }_{1}+{\varepsilon }_{2})w}{{D}_{xx}}{(\frac{2\pi c}{{\lambda }_{p}})}^{2}$$

**Figure 3:**
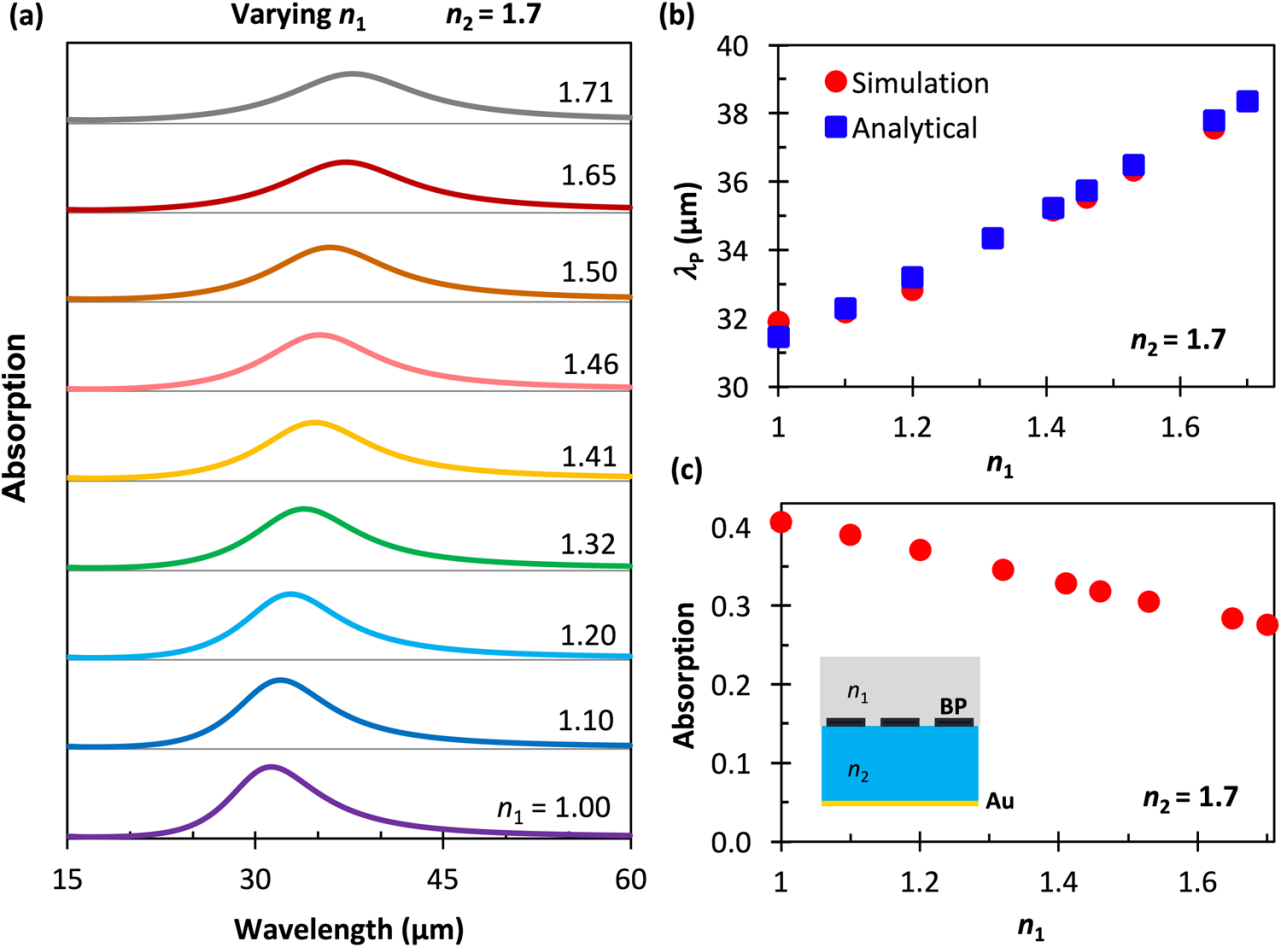
Calculated results showing the effects of varying *n*_1_ on the absorption spectrum with *n*_2_ held constant. **(a)** Simulated normal-incidence TM mode electric field absorption spectra for BP nanoribbons surrounded by different materials with refractive indices, *n*_1_, on a substrate with *n*_2_ = 1.7 (Al_2_O_3_) and a BP electron density of *N* = 10^13^ cm^−2^. (b) Absorption peak resonant wavelength of the fundamental mode (*m* = 1) with respect to the refractive index of the surrounding dielectric layer (*n*_1_). The red dots are from the FEM simulations, and the blue dotes are calculated from the theoretical model described in Eq. (4). (**c**) Peak absorption amplitude as a function of *n*_1_.

Figure 2(b and c) illustrate the absorption resonance wavelength for different values of *n*_2_ obtained both via simulation and theoretical calculation via Eq (5). It is found that primary mode red shift linearly as the dielectric constant increases of the resonance wavelength consistent result in both theory and simulation. To further elucidate the effect of the surrounding dielectric, Fig. 3(a and b) show calculated and simulated absorption spectra for a range of *n*_1_ from 1 to 1.71 for fixed *n*_2_ = 1.71. The first-order phase factor was calculated using Eq. (5) for ranges between 0.41 π and 0.46 π, depending on the dielectric environment. Although similar situations are observed, the spectral broadening and resonance wavelength shifts are much weaker compared to the case of changing *n*_2_. The absorption peak wavelengths are only for dipolar modes and stay between 27% to 41% with the change of the top dielectric environment, Fig. 3(c). The resonance properties in the absorption spectra intensity and line width are also influenced by the optical loss in the BP ribbons, which is mainly characterized by the real part of the conductivity.

## Mechanisms of Enhancing Absorption

The theoretical model predictions of enhanced infrared absorption and plasmonic resonance depend on the effective mass and density of carriers in the BP ribbons. The wavelength of the plasmon resonance in phosphorene nanoribbons scales proportionally with *N*^−1/2^, the same as in conventional semiconductors but contrary to graphene nanoribbons, which show a proportionality of *N*^−1/4 ^^45^. According to Eq. (5), decreasing the anisotropic effective mass, which is dependent on the BP layer thickness, results in a blueshift and strong plasmon localization, while increasing the electron carrier density causes a redshift. The electron (carrier) density, related to the carrier mobility, is controlled by the chosen type of dielectric interface^58,59^, the introduction of doping, or gated-modulation^21^. To understand the tunability of the plasmon resonance and therefore the absorption wavelength in BP ribbons, it is instructive to inspect the significance of altering the conductivity and the ribbon geometry.

**Figure 4:**
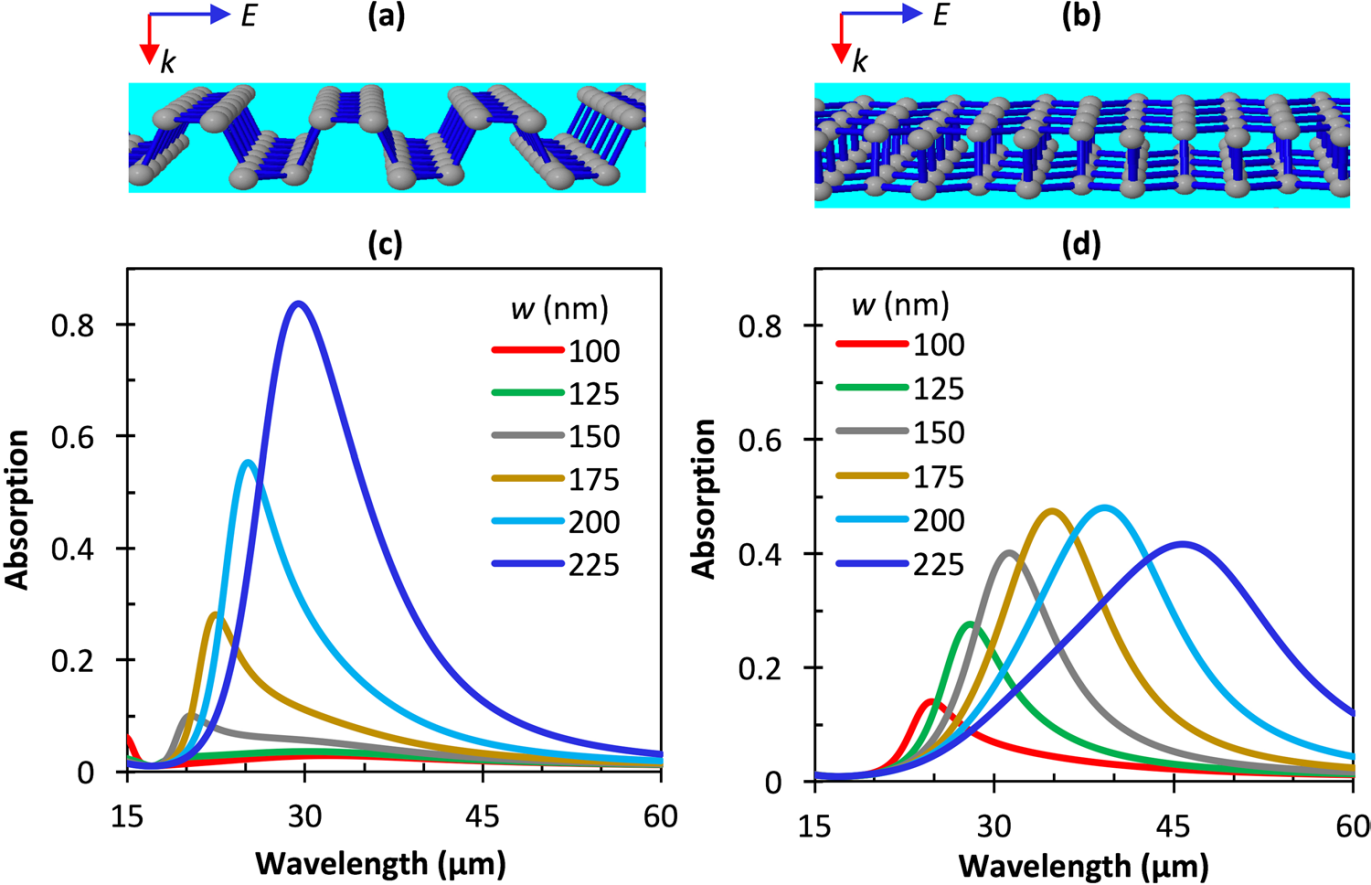
Comparison of the optical response of light polarized in the **(a)** armchair and **(b)** zigzag directions. Simulated absorption spectra for normal-incidence TM mode light polarized along the **(c)** armchair and **(d)** zigzag directions for different *w*. Here, *n*_2_ = 1.71, *n*_1_ = 1.0, *N* = 2.5 × 10^13^ cm^−2^, *P* = 250 nm, and *w* is swept from 100 to 225 nm.

The armchair and zigzag directions of phosphorene are shown in Fig. 4(a and b), respectively. We would like to achieve higher absorption enhancement for light polarized in each direction. Here, optical constants of *n*_1_ = 1.0 and *n*_2_ = 1.71 and a reflective gold layer, as in Fig. 1(b), were considered. According to Eq. (5), one can tune the absorption spectra by changing the number density and the width of BP ribbons. To better quantitatively understand this tunability, absorption simulation results were obtained for the situation where the number density *N* = 5 × 10^12^, 7.5 × 10^12^, and 2.5 × 10^13^ cm^−2^, and the period was constant at *P* = 250 nm over a range of *w* from 100 nm to 225 nm. Figure 4(c and d) show plots of absorption spectra for light polarized in the armchair and zigzag directions, respectively, for the simulated range of *w* values for *N* = 2.5 × 10^13^ cm^−2^. The results for *N* = 5 × 10^12^ and 7.5 × 10^12^ cm^−2^ are provided in supplementary Fig. S1.

The peak absorption wavelength position is highly sensitive to *w* due to the resonance condition of the localized surface plasmons, and it is also affected by the ribbon spacing, *g*(*w*) = *P* − *w*, due to strongly coupled resonances between neighboring ribbons. With a constant period of 250 nm, the gap between ribbons becomes small for large *w*, increasing the strength of the coupling effect. Shown in Fig. 4(c), the absorption is minimal for light polarized in the armchair direction when *w* is small (and the gap is large) ‒ due to weak plasmon localization and less field confinement at the edges. As *w* increases, so does the absorption, up to 0.83 for *w* = 225 nm, corresponding also to the smallest spacing value (*g* = 25 nm), since the coupling between neighboring ribbons increases. For the larger widths, the absorption peak also redshifts as *w* increases due to both width-dependent plasmon resonances and gap-dependent coupling between ribbons. In the zigzag direction, Fig. 4(d), the resonant wavelength redshifts from ~25 μm to 47 μm over the range *w* = 100 – 225 nm. In both the armchair and zigzag orientations, the peak shift shows that the ribbon width and spacing play important roles in tuning the BP ribbon plasmon resonance. The difference in the peak position and amplitude for the same values of *w* between zigzag and armchair directions is due to each atomic orientation having different anisotropic masses, which leads to different imaginary parts of the dielectric function; see Eq. (1) and (2).

The absorption strength in the zigzag direction increases from 0.14 to 0.48 for number density *N* = 2.5 × 10^13^ cm^−2^, compared to the absorption peak value of 0.13 reported by Liu and Aydin for *N* = 10^13^ cm^−2^ ^23^. When one compares the peak wavelengths between *N* = 10^13^ cm^−2^ (Fig. 2) and *N* = 2.5 × 10^13^ cm^−2^ (Fig. 4) for *w* = 150, both armchair and zigzag directions show a blueshift of the absorption peak with increased carrier concentration, consistent with the prediction of Eq. (5). For carrier number density below *N* = 10^13^ cm^−2^ (see supplementary information), a width-dependent-dominant absorption is observed in the armchair direction and a weak resonance in the zigzag direction. Therefore, changing the electron number density, *N*, by using different substrates, doping the BP, or gating introduces a mechanism for tuning and amplifying the plasmonic resonance in BP.

## Encapsulated BP Ribbons

Black phosphorus is highly reactive with oxygen; upon exposure to the environment, it degrades in a matter of minutes or hours^21,31–37^. Also, exposure to moisture causes significant distortion of its structure, causing the formation of porous regions that eventually decompose^31–37^. Encapsulation of BP with a thin dielectric sheet is essential for stability^58,59^. Therefore, we investigate how the dielectric sheet affects the edge plasmon modes in BP. Numerical simulations have been performed to demonstrate the electromagnetic response of phosphorene nanoribbons capped with two materials: a lossless dielectric, Al_2_O_3_, and a hyperbolic metamaterial hexagonal boron nitride (hBN) with optical constants obtained from^60^.

**Figure 5:**
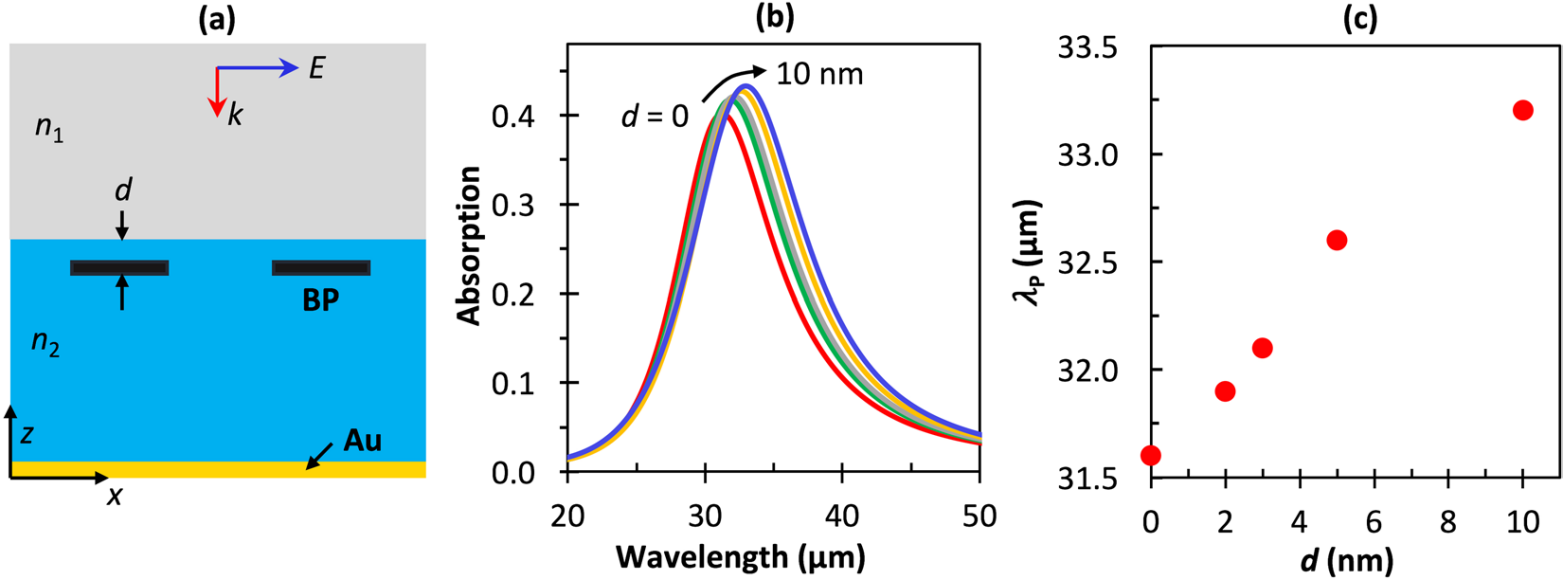
Results for simulated patterned BP nanoribbons encapsulated by a dielectric buffered layer at different depths, *d*. **(a)** 2D cross-sectional schematic **(b)** Simulated absorption spectra for *d* = 0–10 nm and **(c)** resonance peak position as a function of *d*. Here, *P* = 250 nm, *w* = 150 nm, *n*_1_ = 1.0, *N* = 10^13^ cm^−2^, and *n*_2_ = 1.71 (Al_2_O_3_).

First, encapsulation via Al_2_O_3_ was studied, with the results shown in Fig. 5. Figure 5(a) shows the geometry of the model. For consistency with earlier discussions, we kept *P* = 250 nm, *w* = 150 nm, *n*_1_ = 1.0, and *n*_2_ = 1.71, the refractive index of Al_2_O_3_. Figure 5(b) shows the absorption spectra, corresponding to infrared plasmons, with the BP ribbon positioned at different distances (*d*) inside the substrate in the armchair direction. The peak wavelength shifts by approximately 1.5 μm as *d* increases from 0 to 10 nm inside the substrate, shown in detail in Fig. 5(c). The range of *d* is important to maintain the absorption amplitude so that it does not lead to a large change in the optical path length within the dielectric, potentially creating a standing wave. The small shift in wavelength and consistent peak absorption amplitude from Fig. 5 verify that dielectric layer encapsulation helps to conserve the main plasmon resonance properties. These small fine-tuned plasmon wavelength shifts due to a thin capping layer have interesting implications for light-matter interactions with regard to BP plasmon infrared nanoresonators, potentially for highly sensitive sensors.

**Figure 6:**
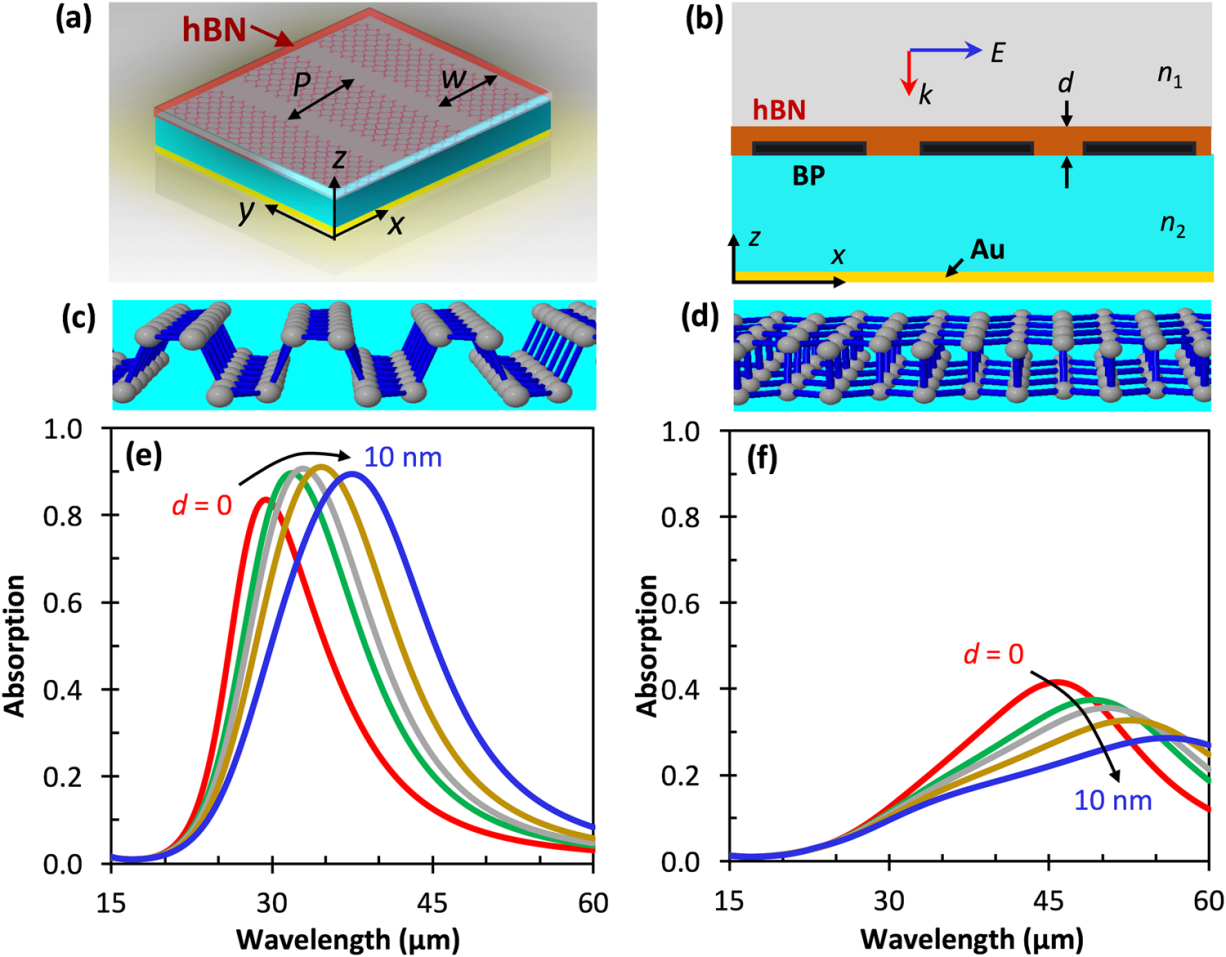
(**a**) 3D and (**b**) 2D schematics of the simulated BP ribbons covered by a protective layer of hBN film of thickness *d*. **(c)** Armchair and **(d)** zigzag polarization directions as shown in Fig. 5. Simulated normal-incidence TM mode electric field absorption spectra for different thicknesses of hBN encapsulating BP for **(e)** armchair and **(f)** zigzag directions. Here, *w* = 225 nm, *P* = 250 nm, *n*_1_ = 1.0, *n*_2_ = 1.71, and *N* = 2.5 × 10^13^ cm^−2^.

Next, we simulated hBN, anisotropic hyperbolic material, as an overlayered sheet for preserving the surface of the BP ribbons from degradation. hBN provides a superior protection layer for BP, mainly because it provides high mechanical strength, high thermal stability and chemical inertness^59,61^. In addition, the layer numbers (thickness) of hBN can be precisely controlled from monolayer to multilayer during the fabrication process through mechanical exfoliation or chemical vapor deposition methods^62^. Figure 6(a) and (b) depict the simulation design for BP ribbons encapsulated with hBN layers of different thickness, *d*. The armchair and zigzag directions are depicted again in Fig. 6(c and d), respectively. The simulated total absorption spectrum with *d* swept from 0 to 10 nm for TM polarized light in the zigzag and armchair directions are shown in Fig. 6(e and f), respectively.

The case of extremely small gaps between ribbons (*w* = 225 nm) was studied for *N* = 2.5 × 10^13^ cm^−2^. As shown in Fig. 6(e and f), the resonant wavelength shifts from 29 μm to 39 μm for armchair polarization, and 47 μm to 56 μm for zigzag. The gradual peak redshift is significantly larger than that of BP embedded in Al_2_O_3_. The absorption intensity is maintained between 80% and 90% in the armchair direction. The zigzag peak absorption drops gradually from 42% to 29% as the thickness of hBN increases, less than half of the values for armchair peak absorption. The presence of the hBN results in a strong localization of the field at the hBN/BP/dielectric interface in armchair direction and weak localization for zigzag. This is mainly due to the full electrical contact and strong interactions in the hybrid interaction that can arise between the ribbon plasmons and a thickness dependent phonon polariton mode that can arise in the hBN^63^. In addition, a noticeable broadening of the absorption peaks occurs for both polarization directions with the introduction of hBN on the BP ribbons.

## Conclusion

In summary, we have investigated propagating surface plasmon properties of black phosphorus sheet- and edge-confined plasmons in surrounding dielectric structures for enhanced tunable absorption. Theoretical schemes of the plasmonic dispersion showed dependence on BP anisotropy, light polarization direction, and dielectric material. In particular, the confinement factor of SPPs has a strong effect, a factor of hundreds, on isolated BP and that increases as the refractive index of the surrounding media increases. Scaling of BP into the nanoribbon size leads to the formation of edge plasmons that trigger enhanced absorption. Simulation results of the spectral position and the absorption peaks can be adjusted both by the anisotropic nature of BP as well as by parameters such as the refractive index, ribbon size, ribbon spacing, and electron density; and the results have validated the theoretical prediction. Additionally, simulations of plasmon enhanced absorption behavior encapsulated the BP ribbon with either a protection nanolayer of lossless dielectric material or the metamaterial hBN to address the possibility of degradation through oxidation. The result shows further mechanisms of tuning resonance modes in infrared wavelengths due to hybridization of BP ribbon edge plasmon and the hyperbolic modes of hBN. The research achievements reveal a promising future for black phosphorene as a plasmonic material with properties that can give a viable platform to plasmon modulated optoelectronic devices across the infrared region of the spectrum.

## Methods

Two-dimensional FEM simulations^51^ were performed to calculate electromagnetic field distributions and absorption spectra on nanoribbon cross-sections that assume infinite length in the *y*-direction. The 2D simulations can accurately approximate calculations of 3D structures so long as the length of the ribbon is large enough compared to the propagation and coupling length of the surface plasmon wavelengths. Periodic boundary conditions were applied along the left and right edges of the model (along the *x*-direction). Perfectly matched layers (PMLs) were added above and below this structure to eliminate the back scattering of electromagnetic waves from the model boundaries. A plane wave polarized in the *x*-direction illuminates the ribbons from above, normal to the substrate surface, in the TM case for the periodic structure. The top boundary was set as the input port and the bottom as the output. A non-uniform mesh was adopted, and the minimum mesh size inside the BP layer equals 0.01 nm, gradually increasing to 50 nm outside the dielectric region.

## Electronic supplementary material

Supplemental Information
