# Supplementary material for: Tuning Infrared Plasmon Resonance of Black Phosphorene Nanoribbon with a Dielectric Interface
Source: Sci Rep. 2018 Feb 19;8:3224. doi: 10.1038/s41598-018-21365-2 (PMC5818661; doi:10.1038/s41598-018-21365-2)
Supplement: Supplementary file 1 — Supplemental Information [file 41598_2018_21365_MOESM1_ESM.pdf]

Supplementary Information

Tuning Infrared Plasmon Resonance of Black Phosphorene Nanoribbon with a Dielectric Interface

Desalegn T. Debu[1,*], Stephen J. Bauman[2], David French[1], Hugh O. H. Churchill[1], and Joseph B. Herzog[1, 2]

[1] University of Arkansas, Department of Physics, Fayetteville, Arkansas 72701, USA

[2] University of Arkansas, Microelectronics-Photonics Graduate Program Fayetteville, Arkansas 72701, USA

[*] dtdebu@uark.edu

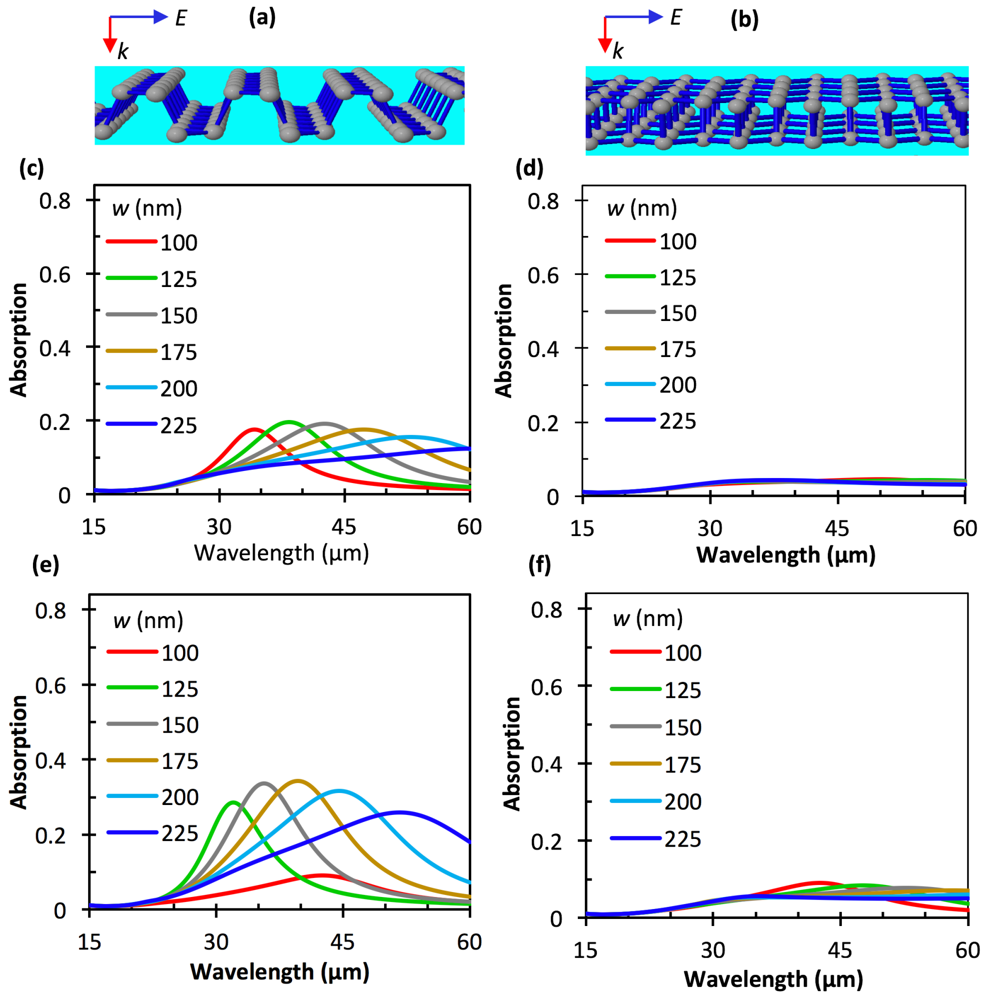

Figure S1. Comparison of the optical response of light polarized in the (a) armchair and (b) zigzag directions. (c) - (f) Simulated absorption spectra for normal-incidence TM mode light polarized along the armchair and zigzag directions for different w: (c) armchair and (d) zigzag for $N = 5\text{x}10^{12}$ cm^{-2}, (e) armchair and (f) zigzag for $N = 7.5\text{x}10^{12}$ cm^{-2} . Here, $n_2 = 1.71$, $n_1 = 1.0$, $P = 250$ nm , and w is swept from 100 to 225 nm.
